# Plasma Matrix Metalloproteinase-9 Levels Predict First-Time Coronary Heart Disease: An 8-Year Follow-Up of a Community-Based Middle Aged Population

**DOI:** 10.1371/journal.pone.0138290

**Published:** 2015-09-21

**Authors:** Peter Garvin, Lena Jonasson, Lennart Nilsson, Magnus Falk, Margareta Kristenson

**Affiliations:** 1 Unit of Research and Development in Local Health Care, County of Östergötland, Linköping, Sweden; 2 Department of Medical and Health Sciences, Division of Community Medicine, Linköping University, Linköping Sweden; 3 Department of Medical and Health Sciences, Division of Cardiovascular Medicine, Linköping University, Linköping, Sweden; Shanghai Institute of Hypertension, CHINA

## Abstract

**Background:**

The enzyme in matrix metalloproteinase (MMP)-9 has been suggested to be an important determinant of plaque degradation. While several studies have shown elevated levels in patients with coronary heart disease, results in prospective population based studies evaluating MMP-9 in relation to first time coronary events have been inconclusive. As of today, there are four published studies which have measured MMP-9 in serum and none using plasma. Measures of MMP-9 in serum have been suggested to have more flaws than measures in plasma.

**Aim:**

To investigate the independent association between plasma levels of MMP-9 and first-time incidence of coronary events in an 8-year follow-up.

**Material and Methods:**

428 men and 438 women, aged 45–69 years, free of previous coronary events and stroke at baseline, were followed-up. Adjustments were made for sex, age, socioeconomic position, behavioral and cardiovascular risk factors, chronic disease at baseline, depressive symptoms, interleukin-6 and C-reactive protein.

**Results:**

53 events were identified during a risk-time of 6 607 person years. Hazard ratio (HR) for MMP-9 after adjustment for all covariates were HR = 1.44 (1.03 to 2.02, p = 0.033). Overall, the effect of adjustments for other cardiovascular risk factors was low.

**Conclusion:**

Levels of plasma MMP-9 are independently associated with risk of first-time CHD events, regardless of adjustments. These results are in contrast to previous prospective population-based studies based on MMP-9 in serum. It is essential that more studies look at MMP-9 levels in plasma to further evaluate the association with first coronary events.

## Introduction

It is well established that inflammation in atherosclerotic plaques plays a major role in development of coronary heart disease (CHD) [1, 2]. Rupture of atherosclerotic plaques is a key event triggering an acute myocardial infarction, and does often occur unexpectedly with a sudden onset.

Several types of cells, in particular macrophages, produce extracellular matrix degrading enzymes, which contribute to the breakdown of the fibrous cap of the plaque [3]. Among matrix degrading enzymes, matrix metalloproteinase (MMP)-9 has been suggested to be an important determinant of plaque degradation [4, 5]. Several groups have reported increased concentrations and activity of MMP-9 in human atherosclerotic plaques with high inflammatory activity, so-called vulnerable plaques [6–9]. In concordance, although the source of MMP-9 cannot be identified when measuring circulating levels, it has been shown repeatedly that levels of circulating MMP-9 are elevated in patients with CHD, in particular those with acute coronary syndrome [3, 10–14]. It has however been frequently discussed whether the levels are elevated prior to onset of disease or elevated due to vascular remodeling following an ischemic event [3, 10–14].

Notably, a number of cross-sectional studies have demonstrated an association between circulating MMP-9 levels and CHD risk factors before onset of disease e.g. hypertension [15, 16], smoking [17, 18], alcohol [18, 19], low physical activity [18], low intake of fruit and vegetables [18] and psychosocial risk factors [20]. Taken together, there is a substantial plausibility that MMP-9 has a role in the progression of a first time CHD event. There is however only a few studies evaluating circulating levels of MMP-9 prospectively in community-based population samples.

A recent meta-analysis including four studies [21–24] demonstrated a non-significant association of MMP-9 levels with CHD incidence, with an aggregated hazard ratio (HR) of 1.07 (0.97–1.19) per SD increment after multivariable adjustments for conventional vascular risk factors [21]. Only one of the four studies in the meta-analysis, from the Danish Research Centre for Prevention and Health cohorts, showed a significant association between MMP-9 and CHD events, with an independent HR of 1.21 (1.05–1.39)[21]. Importantly, all these studies measured MMP-9 in serum samples. Several research groups have shown considerable discrepancies between levels of MMP-9 in serum and plasma, prepared from the same blood draw [25–29]. There are several concerns pointed out, the major one being that pre-analytical preparation of serum causes a release of MMP-9 by neutrophils, which may lead to artificially high levels.

The aim of this study was to investigate the independent association between plasma levels of MMP-9 and incidence of first time CHD in an 8-year follow-up study of a well-characterized Swedish community-based cohort.

## Material and Methods

### Study population

The longitudinal study “Life conditions, Stress and Health” (LSH), has a prospective design and aims at investigating the relationship between socioeconomic status, psychosocial characteristics and risk of CHD, and psychobiological pathways that might mediate these associations. The study population was drawn from a random sample aged 45–69 years, from the County of Östergötland in South East Sweden (n = 898). Exclusion criteria were serious physical or psychiatric illnesses (such as terminal cancer or psychosis). Data collection was conducted in late 2003 and early 2004, and the response rate for the invitation to participate was 62.5%. The Regional Ethical Review Board in Linköping approved the study design (02–0324). All participants provided written informed consent. Six participants did not approve to link their data to the Swedish National Registry of Hospital Admissions, and were excluded from further analyses.

### Biochemical analyses

Venous blood were drawn in the morning in a fasting state, and centrifuged within fifteen minutes to separate plasma, which then was stored in -70°C until analyzed. Concentrations of MMP-9 were measured in EDTA-plasma by human Biotrak ELISA systems (Amersham Biosciences, Uppsala, Sweden). The assay for MMP-9 measures MMP-9, Pro-MMP-9 and the ProMMP-9/TIMP-1 complex. The lower detection limit was 0.6 ng/mL, interassay coefficient of variance (CV) was 7.2 to 7.9%. The analyses were made in two sets. For the first (n = 393), aliquots of plasma were stored for approximately 18 months before laboratory analysis. For the second (n = 488), aliquots were stored for approximately 60 months before laboratory analysis

C-reactive protein (CRP) was measured in serum by a highly sensitive latex-enhanced turbidimetric immunoassay (Roche Diagnostics GmbH, Vienna, Austria) with a lower detection limit of 0.03 mg/L and CV of 1.7%. Aliquots of serum (0.5 mL) were stored in -70°C for approximately 18 months before laboratory analysis.

Levels of interleukin-6 (IL-6) was measured in EDTA-plasma using ultrasensitive bead kit technology (Invitrogen Co., Carlsbad, CA, USA) on a Luminex® 100^TM^ system (Austin, TX, USA). The lower detection limits were set at 1.68 pg/mL and CV was 7.0%. Aliquots of plasma (0.5 mL) were stored in -70°C for approximately 60 months before laboratory analysis.

### Measures of behavioral and cardiovascular risk factors

During a visit to a primary health care center (PHC), participants underwent a short vital status, including height, weight and blood pressure; measured in the right arm in a sitting position after five minutes of rest, using the mean of second and the third measurement (Omron M5-1 digital). Lipid status (ADVIA 1650) was analyzed directly after sample collection.

Furthermore, participants filled out a set of questionnaires. Educational attainment with four answer categories was used as a proxy for socioeconomic position. Smoking habits were based on self-reports, where participants smoking at least one cigarette per day were defined as smokers. To meet the Hill criterion on temporality [30], participants that reported that they had stopped smoking due to illness within the last five years were also included in the smoker category. Physical activity was assessed by two items combining structured exercise (planned activities such as running or swimming) with physical activity in daily life (such as walking the dog, biking to work) and 4 groups were created, with the highest fulfilling the recommended level of daily physical activity according to American College of Sports Medicine [31, 32]. Questions on alcohol intake were based on the validated Food Frequency Questionnaire adopted from the Swedish Mammography Cohort [33]. Three groups of alcohol intake were defined as low to moderate- (up to 80 g/week), high- (81–160 g/week) and very high- (>160 g/week). Cut-offs were guided by earlier studies of cardiovascular benefits and risk associated with drinking [34]. Those who had quit or markedly reduced drinking within the last five years due to illness were included in the highest category.

Questions on fruit and vegetable intake were adopted from the same questionnaire [33] and data was ordered in three groups (mean ± 1SD).

The Center for Epidemiological Studies depression scale (CES-D) [35] was used to capture depressive symptoms.

### Measures of chronic disease and medications at baseline

Chronic disease at baseline was covered using the question “Have you ever had any other chronic or long term disease diagnosed by a medical doctor?” (Yes/No/Don’t know). Dichotomy variables for each condition were used for myocardial infarction, angina pectoris, stroke and diabetes. A composite measure were used to capture other chronic diseases, aggregating chronic obstructive pulmonary disease, cancer, asthma, allergy, kidney disease, coeliac disease, peptic ulcer, rheumatoid arthritis and psychiatric diagnoses into one measure.

Participants were further instructed to write down all ongoing medications based on prescriptions. These were manually regrouped into different categories based on ATC code. In this study, antihypertensive drugs and use of statins were included as dichotomy variables.

### Follow-up of outcomes

First-time major event of CHD was set as main outcome, defined as non-fatal myocardial infarction (MI), and/or an event of invasive coronary revascularization; defined as percutaneous cardiac intervention (PCI) or coronary artery by-pass graft-surgery (CABG). ICD-10 code I21 was used for non-fatal myocardial infarctions. All occurring deaths were checked, to be able to cross-validate underlying cause of death-diagnoses from I20 to I25 as possible myocardial infarctions.

First-time hospitalization due to cerebral infarction (ICD-10 I63, I64 or G45) was set as a secondary outcome, trying to limit the heterogeneous diagnose of stroke by excluding stroke due to subarachnoid, intracerebral or intracranial hemorrhage. Fatal events, regardless of cause, was also set as a secondary outcome.

A power analysis was performed *a priori*. Based on a true twofold hazard ratio, it was estimated that 50 cases was needed to show significant differences (using two-sided probability value of p ≤0.05) with a statistical power of >80%. Actual incidence in the study population was compared with national incidence in Sweden in the same age groups. Based on the analyses, it was predicted that an 8 year follow-up was needed in order to have at least 50 defined primary outcome cases.

Outcome data after 8 year of follow-up time was obtained from the Cause of Death Registry, and the Registry of Hospital Admissions, both from the Swedish National Board of Health and Welfare. The diagnoses were further cross-validated using the patients’ medical journals.

The date for the PHC visit started the follow-up time for each participant. Last date of follow-up was set to 31 Dec 2011, and follow-up time was truncated by major CHD events or any fatal events. Two participants with exceptionally high levels of MMP-9 (more than 3 SD’s higher than the third value in ascending order) were excluded prior to the analyses. Participants with a prior history of self-reported MI were excluded from the primary analyses on first-time major event of CHD. Likewise, analyses on the secondary outcome cerebral infarction were run excluding participants with a prior history of self-reported stroke at baseline.

### Statistical analysis

A set of Cox proportional hazard models with first-time major CHD event as main outcome was defined to evaluate the association with MMP-9. The models were set to facilitate comparisons with analyses on the Danish Research Centre for Prevention and Health cohorts [21]. Thus models were progressively adjusted for sex, age, blood pressure, smoking, history of diabetes, body mass index, triglycerides, cholesterol, alcohol intake, socioeconomic position, IL-6 and CRP (Levels of IL-6 >20 pg/ml and CRP> 10 mg/l and were omitted from the analyses, as this was considered being markers for ongoing acute infection).

In addition, we also adjusted progressively for physical activity, fruit and vegetable intake, depressive symptoms and other present chronic disease at baseline. Time in freezer was accounted for in all regressions, using a variable for number of months in storage along with a dummy variable indicating first or second subset of MMP-9 quantification. Regression models were run on three different measurements for MMP-9, namely standardized z-scores and continuous values, as well as log-transformed values to compensate for skewness in distribution. The models on first-time major CHD event were made in two runs, both excluding participants with reported stroke at baseline as well as adjusting for reported stroke at baseline. Analyses were then repeated using mortality, stroke and a composite measure of CHD and stroke as outcomes. Proportional-hazards assumptions were tested in all regression models using Kaplan-Meier curves and analyses on scaled Schoenfeld residuals on follow-up time.

A two-sided probability value of p ≤0.05 was considered as statistically significant. Analyses were performed in STATA statistical software, release 11.0 (Stata Corporation, College Station, TX, USA) and IBM SPSS for Windows statistical software, release 21 (IBM Corporation, Armonk, NY, USA).

A file with anonymized data is available from the corresponding author upon request.

## Results

Participants with self-reported MI at baseline had significantly higher levels of MMP-9 (age and sex adjusted SD increment = 0.53, p = 0.025), whereas participants with a history of stroke did not differ significantly in comparison to the other participants. After exclusion for previous self-reported MI or stroke, there were 53 cross-validated first-time CHD cases identified over 6 607 person years (n = 866), corresponding to a mean incidence of 8.0 cases per 1 000 person years. The mean follow-up time was 7.6 years per participant (range 0.4 to 8.2 years).

Of the 53 cases, 30 had a diagnosed non-fatal MI, 7 had a fatal MI (all with ICD I21.9 as cause-of-death, no other diagnose within I20-I25 were reported in this material) and 16 underwent invasive coronary revascularization (11 CABG, 5 PCI) due to symptomatic angina pectoris. During follow-up, there were 26 cases of cerebral infarction and 44 fatal events (7 due to MI, 2 due to cerebral infarction, 22 due to malignancies and 13 due to other causes).

The demographic, clinical and laboratory characteristics of the study population are shown in Table 1, along with associations with MMP-9 for each factor. The mean age was 57 years at inclusion. MMP-9 levels had a range from 4.3 mg/l to 143.3 mg/l. The first subset (n = 386) was analyzed approximately 18 months after inclusion and showed a mean level of MMP-9 of 39.4 mg/l (SD 22.2, IQR 23.7 to 50.9). The second subset was analyzed approximately 60 months (n = 480) after inclusion and showed a mean level of 31.9 mg/l (SD 22.7, IQR 15.5 to 42.2). MMP-9 levels were somewhat skewed to the right (skewness value 1.3) in comparison to normal distribution.

**Table 1:** Descriptive characteristics of the study population, and associations with MMP-9.

	Descriptive statistics			Regression to MMP-9	
Factor	n with data	Mean (SD)	categories n (%)	Coefficient^1^	p-value
sex (number of men)	866	-	428 (49)	-0.34	<0.001
age	866	57.0 (7.1)		-0.01	0.478
socio-economic position (4 cat.)	850	-		-0.10	0.004
9 yrs or less of education			311 (36)		
10–11 yrs or less of education			254 (30)		
12 yrs or less of education			109 (13)		
13 yrs or more of education			176 (21)		
smoking (y/n)	835	-	187 (23)	0.47	<0.001
physical activity (4 cat)	799	-		-0.20	<0.001
Very low physical activity			37 (5)		
Low physical activity			266 (33)		
Moderate level			342 (43)		
High level			154 (19)		
alcohol intake (3 cat)	846	-		0.12	0.038
<80 g/week			685 (81)		
80–160 g/week			78 (9)		
>160 g/week			83 (10)		
fruit and vegetable intake (3 cat)	848	-		-0.18	0.008
<mean-1 SD			121 (14)		
mean ± 1SD			602 (71)		
>mean + 1 SD			125 (15)		
depressive symptoms (CES-D)	810	8.9 (7.8)		0.08	0.020
body mass index (3 cat)	858	-		0.20	0.026
<25.0 kg/m^2^			311 (36)		
25.0 to 30.0 kg/m^2^			373 (44)		
>30.0 kg/m^2^			174 (20)		
systolic blood pressure (mm Hg)	856	134 (20)		0.07	0.042
triglycerides (mmol/l)	860	1.4 (0.8)		0.29	<0.001
total cholesterol (mmol/l)	860	5.6 (1.0)		0.03	0.466
non-HDL cholesterol (mmol/l)	848	3.5 (0.9)		0.01	0.764
HDL cholesterol (mmol/l)	860	1.6 (0.4)		-0.12	0.002
IL-6 (ng/l)	803	2.0 (2.8)		0.11	0.009
CRP (mg/l)	819	1.7 (2.0)		0.26	<0.001
angina pectoris at baseline (y/n)	866	-	27 (3)	0.35	0.086
diabetes at baseline (y/n)	866	-	50 (6)	0.24	0.126
other chronic disease (y/n)	866	-	372 (42)	0.11	0.014
statin use (y/n)	866	-	28 (3)	0.13	0.446
antihypertensive drugs (y/n)	866	-	95 (11)	0.19	0.076

^1^ Regression adjusted for age and sex. Coefficient expressed as SD increment of MMP-9 per category or SD increment of continuous variables.

As shown in Table 1, the factors with the largest significant coefficients in the regression models for MMP-9 were smoking (0.47 SD increment, p<0.001) and sex (women 0.34 SD lower than men, p<0.001). This corresponds to differences of +11.8 mg/ml and -8.5 ng/ml, respectively. Out of the 21 factors showed in Table 1, 14 were significantly associated with MMP-9 (all p<0.05). Factors with non-significant associations with MMP-9 were age, total cholesterol, non-HDL cholesterol levels, angina pectoris at baseline, diabetes at baseline, use of statins and antihypertensive drugs.

Participants with first-time coronary event during follow-up had significantly higher levels of MMP-9 at baseline than participants without events (43.2 mg/ml vs 34.5 mg/ml, p = 0.006). Table 2 displays Cox regressions of MMP-9 in relation to first time CHD incidence. The progressive adjustments were done in the same order as the analyses on the Danish Research Centre for Prevention and Health cohorts [21], to facilitate comparisons. The full model adjusting for all covariates had a HR of 1.44 (95% CI 1.03 to 2.02, p = 0.033). The proportional-hazards assumptions were not violated in any of the regression models (p = 0.49 in full model). Neither stroke or angina pectoris at baseline nor statins or antihypertensive drugs had any impact on the association.

**Table 2:** Cox regressions of MMP-9 in relation to CHD incidence with progressive adjustments.

Adjustment^1^	HR (95% CI)^2^	p-value	Other significant variables in the model
Adjustment for sex	1.52 (1.16, 1.98)	0.002	-
plus age	1.55 (1.18, 2.03)	0.001	-
plus systolic blood pressure (SBP)	1.51 (1.15, 1.99)	0.002	-
plus smoking	1.36 (1.02, 1.82)	0.032	smoking (p = 0.004)
plus presence of diabetes	1.37 (1.03, 1.84)	0.029	smoking (p = 0.004)
plus body mass index	1.38 (1.03, 1.86)	0.028	smoking (p = 0.004)
plus triglycerides	1.34 (0.99, 1.81)	0.053	smoking (p = 0.006)
plus total cholesterol	1.37 (1.02;1.86)	0.035	smoking (p = 0.003), total cholesterol (p = 0.004)
plus non-HDL cholesterol^3^	1.40 (1.03, 1.91)	0.029	smoking (p = 0.004), Non-HDL cholesterol (p<0.001)
plus HDL cholesterol^3^	1.41 (1.03, 1.94)	0.030	SBP (p = 0.033), smoking (p = 0.010), Non-HDL cholesterol (p<0.001)
plus alcohol intake^3^	1.40 (1.02, 1.94)	0.034	SBP (p = 0.031), smoking (p = 0.020), Non-HDL cholesterol (p<0.001)
plus socioeconomic position^3^	1.44 (1.04, 1.99)	0.026	SBP (p = 0.026), smoking (p = 0.016), Non-HDL cholesterol (p<0.001)
plus IL-6^3^	1.43 (1.04, 1.98)	0.027	SBP (p = 0.026), smoking (p = 0.015), Non-HDL cholesterol (p<0.001)
plus CRP^3^	1.38 (1.00, 1.93)	0.049	SBP (p = 0.029), smoking (p = 0.014), Non-HDL cholesterol (p<0.001)
plus physical activity^3^	1.41 (1.01, 1.98)	0.039	SBP (p = 0.033), smoking (p = 0.010), Non-HDL cholesterol (p<0.001)
plus depressive symptoms	1.44 (1.03, 2.01)	0.030	SBP (p = 0.038), smoking (p = 0.017), Non-HDL cholesterol (p<0.001)
plus fruit and vegetable intake^3^	1.44 (1.03, 2.01)	0.030	SBP (p = 0.038), smoking (p = 0.022), Non-HDL cholesterol (p<0.001)
plus other severe chronic disease	1.44 (1.03, 2.02)	0.033	SBP (p = 0.040), smoking (p = 0.017), Non-HDL cholesterol (p = 0.001)

^1^ Presented in the same order as the analyses on the Danish Research Centre for Prevention and Health cohorts [21] to facilitate comparison. The approach is identical from adjustment for sex to adjustment for CRP. Progressive adjustments from physical activity and onwards are additional in comparison.

^2^ HR expressed per SD increment of MMP-9. Analyses restricted to participants with information on all variables. 4,944 person years and 35 major CHD events.

^3^ Non-HDL cholesterol is used; total cholesterol omitted, due to co-linearity.

In addition to MMP-9, there were three other factors independently associated with CHD incidence, namely: non-HDL cholesterol (HR per SD = 1.91 95% CI 1.41 to 3.04, p<0.001), smoking (HR = 2.71 95% CI 1.19 to 6.17, p = 0.017) and systolic blood pressure (HR per SD = 1.41 95% CI 1.17 to 1.96, p = 0.040).

Regression models using z-scores, continuous or log-transformed measures on the biomarkers all showed similar results. In full models, continuous levels of MMP-9 corresponds to a HR of 1.39 per SD increment (1.00 to 1.93, p = 0.049), and log-transformed values to HR = 1.51 (1.01 to 2.33, p = 0.044).

Table 3 shows the same set of cox regressions as table 2, using a composite measure of first-time CHD incidence and first-time cerebral infarction. The HR’s for the composite measure are in general about 0.1 lower per SD increment than corresponding HR’s for CHD incidence, in a range from HR = 1.20 to HR = 1.43. The fully adjusted model has a HR of 1.33 (p = 0.058).

**Table 3:** Cox regressions of MMP-9 in relation to a composite measure of CHD-events and cerebral infarction.

	Composite measure of CHD-events and cerebral infarction^1^	
Outcome	HR (95% CI)^1^	p-value
Adjustment for sex	1.41 (1.12, 1.80)	0.003
plus age	1.43 (1.13, 1.82)	0.002
plus systolic blood pressure	1.39 (1.10, 1.76)	0.005
plus smoking	1.26 (0.98, 1.61)	0.072
plus presence of diabetes	1.25 (0.97, 1.61)	0.077
plus body mass index	1.25 (0.97, 1.63)	0.081
plus triglycerides	1.20 (0.92, 1.57)	0.160
plus total cholesterol	1.23 (0.94, 1.60)	0.123
plus non-HDL cholesterol^2^	1.24 (0.94, 1.63)	0.113
plus HDL cholesterol^2^	1.23 (0.93, 1.63)	0.131
plus alcohol intake^2^	1.23 (0.93, 1.63)	0.141
plus socioeconomic position^2^	1.28 (0.96, 1.71)	0.082
plus IL-6^2^	1.28 (0.96, 1.71)	0.081
plus CRP^2^	1.29 (0.97, 1.73)	0.080
plus physical activity^2^	1.31 (0.98, 1.76)	0.064
plus depressive symptoms^2^	1.32 (0.99, 1.77)	0.057
plus fruit and vegetable intake^2^	1.32 (0.99, 1.77)	0.057
plus other severe chronic disease^2^	1.33 (0.99, 1.79)	0.058

^1^ HR expressed per SD increment of MMP-9. Analyses restricted to participants with information on all variables. 4,900 person years and 50 major CHD events or cerebral infarction.

^2^ Non-HDL cholesterol is used; total cholesterol omitted, due to co-linearity.

A similar approach was used for cerebral infarction and mortality as outcomes. The HR’s for cerebral infarction are all non-significant, regardless of adjustment, varying from HR = 1.09 to HR = 1.36 (data not shown). Likewise, the HR’s for total mortality are all non-significant and close to 1.0, regardless of adjustment, varying from HR = 0.95 to HR = 1.03 (data not shown).

As the number of events was low for the latter two, additional analyses were performed adjusting for one single factor at the time, along with age and sex (thereby increasing number of included participants and number of person-years). 24 events of cerebral infarction and 43 fatal events were evaluated (2 events of cerebral infarction and 1 death could not be included due to missing data). All HR’s for MMP-9 were non-significant in those regressions, ranging from HR = 0.97 to 1.11 for cerebral infarction and from HR = 0.90 to 0.97 for fatal events (data not shown).

## Discussion

The main finding of this study was the significant association between plasma MMP-9 and first-time CHD-incidence, regardless of adjustments. As far as we know, this is the first prospective follow-up of first time CHD-events in a community based sample evaluating MMP-9 levels in plasma. Earlier studies have either focused on outcome in a group of patients, or used serum levels of MMP-9. Blankenberg et al. have shown that plasma levels of MMP-9 is associated with cardiovascular death in a group of patients with cardiovascular disease [10]. Kaptoge et al have shown that levels of MMP-9 in serum is associated with cardiovascular events in a population based study [21].

In the other prospective analyses on levels of MMP-9, It has been suggested that MMP-9 has a univariate but not independent association with MI, due to strong associations with other cardiovascular risk factors, in particular with smoking and CRP [22]. It is therefore of note that levels of MMP-9 remain significantly associated with CHD incidence after adjustments in our study. A large number of demographic, clinical and laboratory variables were associated with levels of MMP-9, which is in line with previous studies [15–20]. However, despite considerable associations for MMP-9, including smoking and CRP, the adjustments had only a small effect on the hazard ratios. This implies that MMP-9 is not just a downstream biomarker of conventional risk factors, but may be of clinical relevance as it contributes to CHD risk also via other pathways. Several other groups have investigated possible mechanisms linking MMP-9 to major CHD events [4, 5]. In short, MMP-9 has been shown to be up-regulated in inflammation. The enzyme has specificity to collagen types in basal lamina (inparticual collagen type IV), Thus, high levels of MMP-9 may contribute to vulnerability of atherosclerotic plaques, making them more prone to rupture [4, 5]. Moreover, it has been shown in experimental settings that levels of MMP-9 are influenced by antioxidant/oxidant imbalance [36, 37], and stress hormones [38]. This, along with genetic disposition and expression should be further investigated.

The differences in findings compared to existing literature are probably not, to any major extent, explained by different approaches when adjusting for covariates, as all studies have used extensive adjustments. In this light, the discrepancies in MMP-9 levels that may occur due to varying pre-analytical treatment of sera and plasma should be considered [25–28]. This may create a pitfall in interpretations of circulating levels of MMP-9. It has been shown that the problem is not only a matter of artificially high levels due to release in preparation of serum, but also due to a poor correlation between MMP-9 in serum and plasma. For instance, Jung et al have demonstrated that the elevated levels in serum in comparison to plasma varies from about 3-fold to about 20-fold within the same study [27]. Moreover, the poor correlation between levels in plasma and serum has been demonstrated in a group of patients with renal cell carcinoma, in which MMP-9 levels were significantly higher compared to controls when plasma was analyzed, but significantly lower when serum from the same blood draw was analyzed [25].

Another issue that may need attention when evaluating the prospective association between levels of MMP-9 and CHD incidence is that all previous results are based on samples that have been kept in a freezer for a relatively long time before analysis, ranging in mean duration from seven [22] to twenty-two years [21]. It has been demonstrated that there is a considerable degradation of MMP-9 over time even when kept in -80 degrees [39]. The second set of MMP-9 analyses in our study (analyzed approximately 60 months after blood draw) showed levels that were about 80% in comparison to the first set of analyses (analyzed approximately 18 months after blood draw). Since both subsets are random sample from the study population, this brings further support for a degradation effect during storage. It is today not possible to determine whether the degradation has any effect on the association between MMP-9 and CHD, but it renders further investigation. On one hand, our samples have a shorter storage time compared with other studies but on the other hand, they have been stored for a considerable time. A post-hoc analysis in our material suggested a significant association between MMP-9 and CHD events in the second but not the first subset. The first subset cannot however be properly evaluated due to very few events (n = 11) in that group.

Further, it should be noted that levels of MMP-9 varies considerably between studies [21–24]. Our plasma levels are in the same range as those reported in serum from the Danish Research Centre for Prevention and Health cohorts [21]. Jefferis and colleagues report MMP-9 levels in serum that are about tenfold higher [22]. On the other hand, Hansson and colleagues report mean levels in serum that is about 1% of what is found in our study [23]. The differences is likely caused by a combination of different approaches in pre-analytical treatment [25, 26], variations in time in storage [39] as well as variations of properties over different ELISA kits and batches and highlight difficulties when comparing different studies. For this reason, we choose to present Hazard ratio’s in relation to SD increment rather than absolute levels of MMP-9.

The association between MMP-9 and total mortality was low, possibly due to the relatively low death rates in this age group, with a large variation in cause-of-death. We might however speculate that MMP-9 is of higher relevance as a specific biomarker for CHD events, than as a predictive biomarker for all-cause mortality. The association between MMP-9 and cerebral infarction was also low, but were analyzed with few events during follow-up.

To increase the number of events, we used a composite measure of CHD events and cerebral infarction. The Hazard Ratios for the composite measure are somewhat lower than for CHD incidence when evaluated as its own entity suggesting that MMP-9 is a more relevant marker for CHD incidence than cerebral infarction. Thus, composite measures including both CHD events and stroke should be used with caution when evaluating MMP-9 as a predictive marker.

### Limitations and strengths

The major limitation in this study is the low number of cases during follow-up. This is further accentuated in full regression models, due to with missing data on at least one of the factors adjusted for. We chose to include a large number of dependent variables to facilitate comparisons with the published meta-analysis [21], and to adjust for other well-known risk factors that wasn’t covered in the meta-analysis. We are well aware that the full regression models include more independent variables than is typically the case when evaluating an association based on a low number of cases.

However and importantly, as the hazard ratio’s remain close to the crude hazard ratio regardless of adjustments, we don’t have indications neither of severe over-fitting nor under-fitting due to many variables in the models. On the contrary, there is low probability that our main findings are caused by a few outliers in the study population, as there is robustness in the regression analyses, with significant hazard ratios regardless if z-scores, continuous, or log-transformed measures were used for MMP-9 levels. The validity of our data is also supported by the consistency in the regression models where proportional hazard-assumptions hold regardless of adjustment.

The possibilities to conduct analyses of prediction ability (such as C-statistics or ROC comparisons) in this material are limited by the low number of cases during follow-up. This is illustrated by the presumably large rate of beta errors in the regression analyses in table 2. However, this does not detract from our primary aim to evaluate MMP-9 in plasma rather than serum. Evaluation of the net effect of MMP-9 measures in prediction ability of the models *per se* to discuss potential improvement in risk stratification accuracy should be performed in a material with a larger number of events.

### Future perspective

High levels of MMP-9 may contribute to vulnerability of atherosclerotic plaques, making them more prone to rupture. Previous analyses in serum rather than plasma may have biased the view upon the usefulness of MMP-9 as a predictor. It is essential that more studies look at MMP-9 levels in plasma to evaluate the association with new first coronary events. We might speculate that this would be done in two steps. First our results based on MMP-9 in plasma rather than serum should be confirmed or contradicted by other existing biobanks together with longitudinal data on onset of first time coronary events. Second, analyses of prediction ability should be conducted in this study as well as other studies.

## Conclusions

In a middle-aged population free of CHD, plasma MMP-9 was independently associated with incidence of CHD events during an 8-year follow-up. Our findings demonstrate that plasma levels of MMP-9 prior to onset of first-time coronary heart events are associated with risk for an event during follow-up. The significant association based on plasma levels differs from a recently published meta-analysis on MMP-9 in serum and coronary events.

## Supporting Information

S1 File All relevant data for the analysis can be found in the uploaded data set, provided in SPSS-format. (SAV)
